# Animal Reservoirs and Hosts for Emerging Alphacoronaviruses and Betacoronaviruses

**DOI:** 10.3201/eid2704.203945

**Published:** 2021-04

**Authors:** Ria R. Ghai, Ann Carpenter, Amanda Y. Liew, Krystalyn B. Martin, Meghan K. Herring, Susan I. Gerber, Aron J. Hall, Jonathan M. Sleeman, Sophie VonDobschuetz, Casey Barton Behravesh

**Affiliations:** Centers for Disease Control and Prevention, Atlanta, Georgia, USA (R.R. Ghai, A. Carpenter, A.Y. Liew, K.B. Martin, M.K. Herring, S.I. Gerber, A.J. Hall, C. Barton Behravesh);; Emory University, Atlanta (A.Y. Liew, K.B. Martin, M.K. Herring); US Geological Survey, Madison, Wisconsin, USA (J.M. Sleeman);; Food and Agriculture Organization of the United Nations, Rome, Italy (S. VonDobschuetz)

**Keywords:** coronaviruses, viruses, animal reservoirs, hosts, coronavirus disease, COVID-19, severe acute respiratory syndrome coronavirus 2, SARS-CoV-2, Middle East respiratory syndrome coronavirus, MERS-CoV, respiratory infections, zoonoses

## Abstract

The ongoing global pandemic caused by coronavirus disease has once again demonstrated the role of the family *Coronaviridae* in causing human disease outbreaks. Because severe acute respiratory syndrome coronavirus 2 was first detected in December 2019, information on its tropism, host range, and clinical manifestations in animals is limited. Given the limited information, data from other coronaviruses might be useful for informing scientific inquiry, risk assessment, and decision-making. We reviewed endemic and emerging infections of alphacoronaviruses and betacoronaviruses in wildlife, livestock, and companion animals and provide information on the receptor use, known hosts, and clinical signs associated with each host for 15 coronaviruses detected in humans and animals. This information can be used to guide implementation of a One Health approach that involves human health, animal health, environmental, and other relevant partners in developing strategies for preparedness, response, and control to current and future coronavirus disease threats.

Coronaviruses are a family of RNA viruses whose large genomes, propensity for mutation, and frequent recombination events have resulted in a diversity of strains and species that are capable of rapid adaptation to new hosts and ecologic environments (*1*). This viral plasticity has garnered widespread concern because of zoonotic potential and the consequences of new emergence events in both human and animal populations. The emergence of a new strain of severe acute respiratory syndrome coronavirus 2 (SARS-CoV-2), which causes coronavirus disease (COVID-19) has once again demonstrated the role of the family *Coronaviridae* in causing human disease outbreaks. SARS-CoV-2, a novel betacoronavirus, was identified in human patients from Wuhan, China, during December 2019 and has resulted in a global pandemic, an unprecedented public health emergency, and untold economic and societal repercussions worldwide. Similar to the 2002–2003 severe acute respiratory syndrome (SARS) epidemic, a live animal market where hundreds of animal species were sold is suspected to be associated with the emergence or early spread of COVID-19 in humans (*2*).

Although COVID-19 is novel in the breadth of the human outbreak, several pathogenic alphacoronaviruses and betacoronaviruses have shown similar patterns of emergence. As early as the 1930s, coronaviruses pathogenic to livestock, companion animals, and laboratory animals were identified (*3*). During the 1960s, 2 human coronaviruses, HCoV-229E and HCoV-OC43, were detected in patients who had common colds (*4*,*5*). Although it is speculated that HCoV-OC43 might also have emerged through a global pandemic in the late 1800s (*6*), the 2002–2003 SARS outbreak is the first known global epidemic caused by a coronavirus. The SARS epidemic triggered research within this viral family (*3*). This research led to detection of 2 new human coronaviruses, HCoV-NL63 and HCoV-HKU1 (*7*,*8*). HCoV-229E, HCoV-OC43, HCoV-NL63, and HCoV-HKU1 are now accepted as globally endemic common cold species that are typically associated with mild-to-moderate respiratory illness. In 2012, the most deadly human coronavirus to date was detected in the Arabian Peninsula: Middle East respiratory syndrome coronavirus (MERS-CoV) (*9*). A cumulative body of research on these and other coronaviruses has shown that most alphacoronaviruses and betacoronaviruses infecting humans have come from animal hosts and that both historic patterns and coronavirus biology establish an urgent ongoing threat to human and animal health (*10*). Although coronaviruses are divided into 4 viral genera, namely alphacoronaviruses, betacoronaviruses, gammacoronaviruses, and deltacoronaviruses, we focus on alphacoronaviruses and betacoronaviruses because all known human coronaviruses are from these genera, and they may therefore pose an increased risk for causing future pandemics.

This review is intended to compile data to inform a One Health approach to combatting emerging alphacoronaviruses and betacoronaviruses. One Health is a collaborative, multisectoral, and transdisciplinary approach—working at the local, regional, national, and global levels—with the goal of achieving optimal health outcomes recognizing the interconnection between humans, animals, plants, and their shared environment (*11*). For example, in Qatar, a One Health approach for MERS-CoV prevention and control has been implemented since early in the outbreak, and is associated with improvements in coordination, joint outbreak response rates, and diagnostic capacity (*12*). Similarly, in the United States, establishment of the One Health Federal Interagency COVID-19 Coordination Group has been instrumental in ensuring an efficient and coordinated all-of-government response by creating a mechanism to communicate, share timely updates, and align messaging (*13*). More generally, the One Health approach is endorsed as an effective means of combatting zoonotic diseases internationally by the Tripartite international health organizations, consisting of the Food and Agriculture Organization of the United Nations, the World Health Organization, and the World Organisation for Animal Health (*14*).

As with other zoonotic diseases, effective implementation of a One Health approach for emerging coronaviruses requires an understanding of the transmission dynamics and human and animal hosts associated with the pathogen. Therefore, this review summarizes information from other coronavirus emergence events, which might be useful in identifying trends, establishing baselines, and informing decision-making by using a One Health approach around the current COVID-19 pandemic and future emerging coronavirus threats. Specifically, we provide information on the receptor used by each current or previously emerging coronavirus because tropism can help predict host susceptibility (Table 1) for all known hosts of each coronavirus and their host category (i.e., reservoir, intermediate, spillover, susceptible through experimental infection, or nonsusceptible through experimental infection) (Table 2) and clinical signs associated with coronavirus infection (Table 3)

**Table 1:** Current or previously emerging coronaviruses*

Pathogen (abbreviation)	Disease (abbreviation)	Viral genus	Receptor (abbreviation) [suspected]
Alphacoronavirus 1 (ACoV1); strain canine enteric coronavirus (CCoV)	Canine coronavirus infection (CCoV)	*Alphacoronavirus*	Aminopeptidase N (APN, CD13)
Alphacoronavirus 1 (ACoV1); strain feline infectious peritonitis virus (FIPV)	Feline infectious peritonitis virus (FIP)	*Alphacoronavirus*	Aminopeptidase N (APN, CD13)
Bat coronavirus HKU10	NA	*Alphacoronavirus*	Unknown
Ferret systemic coronavirus (FRSCV)	Ferret systemic coronavirus (FRSCV)–associated disease	*Alphacoronavirus*	Unknown
Human coronavirus NL63	Common cold	*Alphacoronavirus*	Angiotensin-converting enzyme 2 (ACE2)
Human coronavirus 229E	Common cold	*Alphacoronavirus*	Human aminopeptidase N (hAPN, CD13)
Porcine epidemic diarrhea virus (PEDV)	Porcine epidemic diarrhea (PED)	*Alphacoronavirus*	[Aminopeptidase N (APN, CD13)]
Rhinolophus bat coronavirus HKU2; strain swine acute diarrhea syndrome coronavirus (SADS-CoV)	Swine acute diarrhea syndrome (SADS)	*Alphacoronavirus*	Unknown
Betacoronavirus 1; strain bovine coronavirus	NA	*Betacoronavirus*	Human leukocyte antigen class I (HLA-1)
Betacoronavirus 1; strain canine respiratory coronavirus	Canine infectious respiratory disease (CIRD)	*Betacoronavirus*	Human leukocyte antigen class I (HLA-1)
Betacoronavirus1; strain human coronavirus OC43	Common cold	*Betacoronavirus*	Human leukocyte antigen class I (HLA-1)
Human coronavirus HKU1	Common cold	*Betacoronavirus*	Human leukocyte antigen class I (HLA-1)
Middle East respiratory syndrome coronavirus (MERS-CoV)	Middle East respiratory syndrome (MERS)	*Betacoronavirus*	Dipeptidyl peptidase 4 (DPP4, CD26)
Severe acute respiratory syndrome coronavirus (SARS-CoV)	Severe acute respiratory syndrome (SARS)	*Betacoronavirus*	Angiotensin-converting enzyme 2 (ACE2)
Severe acute respiratory syndrome coronavirus 2 (SARS-CoV-2)	Coronavirus disease (COVID-19)	*Betacoronavirus*	Angiotensin-converting enzyme 2 (ACE2)

*All coronaviruses are described in Tables 2 and 3, including the receptor used for viral entry. NA, not available.

**Table 2:** Hosts and reservoirs of current or previously emerging coronaviruses*

Pathogen (abbreviation)	Reservoir host [suspected]	Intermediate/amplifying hosts(s) [suspected]	Spillover host(s)	Suspected susceptible hosts	Nonsusceptible host(s)
Alphacoronavirus1 (ACoV1); strain canine enteric coronavirus (CCoV)	Domestic dog (*Canis lupus familiaris*)	Unknown/none identified	Carnivores; family Canidae	Unknown/none identified	Unknown/none identified
			Carnivores; family Felidae		
			Carnivores; family Mustelidae		
			Carnivores; family Viverridae		
Alphacoronavirus1 (ACoV1); strain feline infectious peritonitis virus (FIPV)	Domestic cat (*Felis catus*)	Unknown/none identified	Carnivores: family Felidae	Mouse (*Mus musculus*)	
Bat coronavirus HKU10	Leschenault’s rousettes bat (*Rousettus leschenaulti*)	Unknown/none identified	Pomona leaf-nosed bats (*Hipposideros pomona)*	Unknown/none identified	Unknown/none identified
Ferret systemic coronavirus (FRSCV)	Ferret (*Mustela putorius*)	Unknown/none identified	Ferret (*Mustela putorius)*	Unknown/none identified	Unknown/none identified
Human coronavirus NL63	Bats; family Hipposideridae	Unknown/none identified	Human	Unknown/none identified	Cattle (*Bos taurus*)
					Domestic pig (*Sus scrofa*)
					Donkey (*Equus africanus*)
					Goat (*Capra aegagrus*)
					Sheep (*Ovis aries)*
Human coronavirus 229E	Bats; family Hipposideridae	Dromedary camels (*Camelus dromedarius*)	Alpaca (*Vicugna pacos*)	Domestic cat (*Felis catus*)	
			Human		
Porcine epidemic diarrhea virus (PEDV)	Bats; family Vespertillionidae	Unknown/none identified	Domestic pig (*Sus scrofa*)	Unknown/none identified	Unknown/none identified
Rhinolophus bat coronavirus HKU2; strain swine acute diarrhea syndrome coronavirus (SADS-CoV)	Bats; family Rhinolophidae	Unknown/one identified	Domestic pig (*Sus scrofa*)	Mouse (*Mus musculus*)	Unknown/none identified
Betacoronavirus 1; strain bovine coronavirus	[Rodent; family Muridae]	Unknown/none identified	Cattle (*Bos taurus*)	Turkey (*Meleagris gallopavo)*	Chicken (*Gallus gallus*)
Betacoronavirus 1; strain canine respiratory coronavirus	[Rodent; family Muridae]	[Cattle] (*Bos taurus*)	Domestic dog (*Canis lupus familiaris)*	Unknown/none identified	Unknown/none identified
Betacoronavirus 1; strain uman coronavirus OC43	[Rodent; family Muridae]	Artiodactyl; family Bovidae	Chimpanzees (*Pan troglodytes)*	Mouse (*Mus musculus*)	Unknown/none identified
			Human		
Human coronavirus HKU1	[Rodent; family Muridae]	Unknown/none identified	Human	Unknown/none identified	Unknown/none identified
Middle East respiratory syndrome coronavirus (MERS-CoV)	Bats; family Vespertillionidae	Dromedary camels (*Camelus dromedarius*)	Human	Alpaca (*Vicugna pacos*)	Ferret (*Mustela putorius furo)*
				Common marmoset (*Callithrix jacchus*)	Golden hamster (*Mesocricetus auratus*)
				Domestic pig (*Sus scrofa*)	Horse (*(Equus ferus caballus)*
				Llama (*Lama glama*)	Mouse (*Mus musculus*)
				New Zealand White rabbit (*Oryctolagus cunuculus*)	Sheep (*Ovis aries*)
				Rhesus macaque (*Macaca mulatta*)	
Severe acute respiratory syndrome coronavirus (SARS-CoV)	Bats; family Rhinolophidae	[Masked palm civet] (*Paguma larvata*)	Chinese ferret badger (*Melogale moschata*)	African green monkey (*Chlorocebus aethiops*)	Chicken (*Gallus gallus*)
			Domestic cat (*Felis catus*)	Cynomolgus macaque (*Macaca fascicularis*)	
			Domestic dog (*Canis lupus familiaris)*	Domestic cat (*Felis catus*)	
			Domestic pig (*Sus scrofa*)	Domestic pig *(Sus scrofa*)	
			Human	Ferret (*Mustela putorius*)	
			Racoon dog (*Nyctereutes procyonoides*)	Golden hamster (*Mesocricetus auratus*)	
				Guinea pig (*Cavia porcellus*)	
				Masked palm civet (*Paguma larvata)*	
				Mouse (*Mus musculus*)	
				Rat (*Rattus sp.*)	
				Rhesus macaque (*Macaca mulatta)*	
Severe acute respiratory syndrome–related coronavirus 2 (SARS-related CoV-2)	[Bats: family Rhinolophidae]	Unknown/none identified	Domestic cat (*Felis catus*)	African green monkey (*Chlorocebus aethiops*)	Chicken (*Gallus gallus*)
			Domestic dog *(Canis lupus familiaris*)	Chinese hamster (*Cricetulus griseus*)	Big brown bat (*Eptesicus fuscus*)
			Ferret (*Mustela putorius*)	Chinese tree shrew (*Tupaia belangeri chinensis*)	Cattle (*Bos taurus*)
			Gorilla (*Gorilla gorilla*)	Common marmoset (*Callithrix jacchus*)	Domestic pig (*Sus scrofa*)
			Human	Cynomolgus macaque (*Macaca fascicularis*)	Japanese quail (*Coturnix japonica*)
			Mink (*Neovison vison*)	Domestic cat (*Felis catus*)	Mouse (*Mus musculus*)
			Lion (*Panthera leo*)	Domestic dog (*Canis lupus familiaris*)	Pekin duck (*Anas platyrhinchos*)
			Puma (*Puma concolor*)	Egyptian fruit bat (*Rousettus aegyptiacus*)	Turkey (*Meleagris gallopavo*)
			Tiger (*Panthera tigris*)	Ferret (*Mustela putorius*)	White Chinese geese (*Anser cygnoides*)
				Golden hamster (*Mesocricetus auratus*)	
				Mink (*Neovison vison*)	
				New Zealand white rabbit (*Oryctolagus cunuculus*)	
				Racoon dog (*Nyctereutes procyonoides*)	
				Rhesus macaque (*Macaca mulatta*)	

*All hosts detected for each coronavirus are shown. A reservoir host is a species in which the pathogen endemically circulates and is considered to have coevolved with. An intermediate host is a species that harbors a recent common ancestor of the coronavirus or played a role in the natural selection/adaptation of the virus before its spillover. A spillover host is a nonendemically infected species in which >1 animals are considered to have acquired the virus through natural infection (e.g., exposure an infected conspecific or interspecies transmission). A susceptible host is a species in which >1 animals can become infected with the virus through experimental challenges or otherwise laboratory controlled infection studies. In vitro studies or studies using transgenic animal models to induce susceptibility are not included. A nonsusceptible host is a species in which experimental challenge or otherwise laboratory controlled infection studies have occurred that did not result in viral infection. For all categories, infection is considered detection of viral RNA from host samples or the host mounting a detectable antibody response. Therefore, infection as it is used here does not necessarily imply hosts naturally or experimentally infected with a virus are capable of transmitting the infection to others.

**Table 3:** Clinical manifestations for infections with current or previously emerging coronaviruses*

Pathogen (abbreviation)	Host	Host type	Body system	Clinical manifestation	Vaccine
Alphacoronavirus1 (ACoV1); strain canine enteric coronavirus (CCoV)	Domestic dog	Reservoir	Gastrointestinal	Vomiting, diarrhea, and dehydration	Yes
	Carnivores; family Canidae	Spillover	Unknown	Unknown	No
	Carnivores; family Felidae	Spillover	Gastrointestinal	Mild diarrhea	No
	Carnivores; family Mustelidae	Spillover	Unknown	Unknown	No
	Carnivores; family Viverridae	Susceptible	Unknown	Unknown	No
Alphacoronavirus1 (ACoV1); strain feline infectious peritonitis virus (FIPV)	Domestic cat	Reservoir	Multisystemic	Varies: fever, weight loss, diarrhea, ascites, thoracic effusion, ocular signs, neurologic signs, and death	Yes†
	Carnivores: family Felidae	Spillover	Multisystemic	Weight loss, fever, diarrhea, jaundice	No
Bat coronavirus HKU10	Leschenault’s rousettes bat	Reservoir	NA	Asymptomatic	No
	Pomona leaf-nosed bats	Spillover	Unknown	Lower bodyweight than noninfected bats	No
Ferret systemic coronavirus (FRSCV)	Ferret	Reservoir, spillover	Multisystemic	Anorexia, weight loss, diarrhea, lymphadenopathy, anemia, and neurologic signs	No
Human coronavirus NL63	Bats; family Hipposideridae	Reservoir	NA	Asymptomatic	No
	Human	Spillover	Respiratory	Fever, cough, rhinorrhea	No
Human coronavirus 229E	Bats; family Hipposideridae	Reservoir	NA	Asymptomatic	No
	Dromedary camels	Intermediate	NA	Asymptomatic	No
	Alpaca	Spillover	Respiratory	Acute respiratory signs, death	No
	Human	Spillover	Respiratory	Nasal congestion, rhinorrhea. Can progress to pneumonia.	No
	Domestic cat	Susceptible	NA	Asymptomatic	No
Porcine epidemic diarrhea virus (PEDV)	Bats; family Vespertillionidae	Reservoir	Unknown	Unknown	No
	Domestic pig	Spillover	Gastrointestinal	Vomiting, diarrhea, and death in piglets, diarrhea and agalactia in pregnant sows	Yes‡
Rhinolophus bat coronavirus HKU2; strain swine acute diarrhea syndrome coronavirus (SADS-CoV)	Bats; family Rhinolophidae	Reservoir	NA	Asymptomatic	No
	Domestic pig	Spillover	Gastrointestinal	Severe, acute diarrhea, rapid weight loss, death in piglets	No
	Mouse	Susceptible	NA	Asymptomatic	NA
Betacoronavirus 1; strain bovine coronavirus	Rodent; family Muridae	Reservoir	Unknown,	Unknown	No
	Cattle	Spillover	Respiratory, gastrointestinal	Respiratory signs, diarrhea, anorexia, lethargy	Yes
	Turkey	Susceptible	Gastrointestinal	Diarrhea, reduced weight gain, and enteritis	NA
Betacoronavirus 1; strain canine respiratory coronavirus	Rodent; family Muridae	Reservoir	Unknown	Unknown	No
	Cattle	Intermediate	Unknown	Unknown	No
	Domestic dog	Spillover	Respiratory	Mild cough, nasal discharge, inappetence, bronchopneumonia	No
Betacoronavirus 1; strain human coronavirus OC43	Rodent; family Muridae	Reservoir	Unknown,	Unknown	No
	Artiodactyl; family Bovidae	Intermediate	Unknown, suspected host	Unknown	No
	Chimpanzees	Spillover	Respiratory	Coughing, sneezing	No
	Human	Spillover	Respiratory	Nasal congestion, rhinorrhea. Can progress to pneumonia	No
	Mouse	Susceptible	Neurologic	Anorexia, weight loss, neurologic signs	No
Human coronavirus HKU1	Rodent; family Muridae	Reservoir	Unknown, suspected host	Unknown, suspected host	No
	Human	Spillover	Respiratory	Rhinorrhea, cough, fever	No
Middle East respiratory syndrome coronavirus (MERS-CoV)	Bats; family Vespertillionidae	Reservoir	NA	Asymptomatic	No
	Dromedary camels	Intermediate	Respiratory	Often asymptomatic, or can manifest with fever, nasal discharge	No
	Human	Spillover	Respiratory, multisystemic	Variable: coughing, shortness of breath, fatigue, myalgia, arthralgia, fever, headaches, vomiting, and diarrhea, sore throat and rhinorrhea. Can progress to pneumonia, acute renal failure, multiorgan failure, and death	No
	Alpaca	Susceptible	NA	Asymptomatic	No
	Common marmoset	Susceptible	Respiratory	Pneumonia	No
	Domestic pig	Susceptible	Respiratory	Nasal discharge	No
	Llama	Susceptible	Respiratory	Nasal discharge	No
	Rhesus macaque	Susceptible	NA	Asymptomatic	No
	New Zealand White rabbit	Susceptible	Respiratory	Asymptomatic	No
Severe acute respiratory syndrome coronavirus (SARS-CoV)	Bats; family Rhinolophidae	Reservoir	Unknown,	Unknown	No
	Masked palm civet	Intermediate, susceptible	Respiratory	Fever, lethargy, pneumonia	No
	Chinese ferret badger	Spillover	NA	Asymptomatic	No
	Domestic cat	Spillover, susceptible	NA	Asymptomatic	No
	Domestic dog	Spillover	Unknown	Unknown	No
	Domestic pig	Spillover, susceptible	NA	Asymptomatic	No
	Human	Spillover	Respiratory	Fever, cough, shortness of breath, difficulty breathing, pneumonia	No
	Racoon dog	Spillover	NA	Asymptomatic	No
	African green monkey	Susceptible	NA	Asymptomatic	No
	Cynomolgus macaque	Susceptible	Respiratory	Lethargy, skin rash, and respiratory distress	No
	Ferret	Susceptible	Respiratory	Lethargy, conjunctivitis, weight loss	No
	Golden hamster	Susceptible	NA	Asymptomatic	No
	Guinea pig	Susceptible	NA	Asymptomatic	No
	Mouse	Susceptible	Nonspecific	Asymptomatic or weight loss and dehydration	No
	Rat	Susceptible	NA	Asymptomatic	No
	Rhesus macaque	Susceptible	Nonspecific	Transient fever	No
Severe acute respiratory syndrome coronavirus 2 (SARS-CoV-2)	Bats: family Rhinolophidae	Reservoir	Unknown	Unknown	No
	Domestic cat	Spillover, susceptible	Respiratory	Asymptomatic or ocular discharge and sneezing, severe outcomes in rare instances	No
	Domestic dog	Spillover, susceptible	NA	Asymptomatic	No
	Ferret	Spillover	Gastrointestinal	Unknown	No
	Gorilla	Spillover	Nonspecific	Lethargy, inappetence, cough	No
	Human	Spillover	Respiratory	Fever, cough, shortness of breath or difficulty breathing	No
	Mink	Spillover, susceptible	Gastrointestinal, respiratory	Respiratory and gastrointestinal signs, increased mortality rate	No
	Lion	Spillover	Respiratory	Cough, inappetence, wheezing	No
	Puma	Spillover	Unknown	Unknown	No
	Tiger	Spillover	Respiratory	Cough, inappetence, wheezing	No
	African green monkey	Susceptible	Nonspecific	Asymptomatic or inappetence, anorexia, mildly increased temperature	No
	Chinese hamster	Susceptible	Respiratory	Weight loss, bronchitis, pneumonia	No
	Chinese tree shrew	Susceptible	NA	Asymptomatic	No
	Common marmoset	Susceptible	NA	Asymptomatic	No
	Cynomolgus macaque	Susceptible	Respiratory	Asymptomatic or nasal discharge	No
	Egyptian fruit bat	Susceptible	Respiratory	Asymptomatic or mild rhinitis	
	Ferret	Susceptible	Respiratory	Asymptomatic or fever, cough, inappetence	No
	Golden hamster	Susceptible	Respiratory	Weight loss, lethargy, ruffled fur, hunched posture, tachypnea, pneumonia	No
	New Zealand white rabbits	Susceptible	NA	Asymptomatic	No
	Racoon dog	Susceptible	Nonspecific	Lethargy	No
	Rhesus macaque	Susceptible	Respiratory	Changes in respiratory pattern, piloerection, reduced appetite, pale appearance, hunched posture, dehydration, coughing	No

*Clinical manifestation of disease in all hosts described in Tables 1 and 2 other than nonsusceptible hosts. NA, not available. †Not recommended for administration by the American Academy of Family Physicians.  ‡Conditional license for use.

## Emerging Coronaviruses and Wildlife

More than 70% of zoonotic emerging infectious diseases in humans are caused by pathogens that have a wildlife origin (*11*). Several mammalian orders are now known to host coronaviruses, including carnivores, lagomorphs, nonhuman primates, ungulates and rodents (*3*). However, the attention has focused on Chiroptera (bats), which are hypothesized to be the origin host for all alphacoronaviruses and betacoronaviruses, and therefore all human coronaviruses (Table 2) (*1*,*3*).

After rodents, bats are the second most diverse and abundant mammalian order, comprising 20% of all mammalian biodiversity worldwide. In the past 2 decades, research has intensified to determine why bats harbor more zoonotic diseases than other mammalian taxa, including pathogens that result in high-consequence infectious diseases, such as Ebola and Marburg filoviruses; Nipah and Hendra paramyxoviruses; and SARS-CoV, SARS-CoV-2, and MERS-CoV, emerging in humans (*15*). Behavioral and ecologic traits, such as their gregariousness, sympatry with mixed species assemblages in roosts, and long lifespan relative to size, have been suggested explanations for why bats are reservoirs to many viral pathogens (*15*). Physiologically, bats have comparatively high metabolic rates and typically do not show clinical signs after viral infection. Recently, it has also been shown that bats have several immune characteristics that are unique among mammals and that cumulatively dampen their antiviral responses (*16*). Those factors also probably contribute to their effectiveness as viral reservoirs.

Coronavirus richness and diversity detected in bats far exceeds those of other mammalian orders; >11 of 18 chiropteran families across 6 continents have tested positive for >1 coronavirus species (*3*). A study surveying the diversity of wildlife coronaviruses across global disease hotspots identified 100 distinct viruses, of which 91 were detected in bats (*10*). This study reported that patterns of coronavirus diversity mirrored bat diversity and evolutionary history, reinforcing the idea that bats are the predominant reservoir of zoonotic and emerging coronaviruses (*10*). On the basis of extrapolations made in the same study, Anthony et al. predicted that bats harbor ≈3,204 coronaviruses, most of which remain undetected (*10*). Although much coronavirus diversity remains to be detected, several SARS-like coronaviruses have been detected already in bats, including viruses that use the same human cellular receptor molecule as SARS-CoV and SARS-CoV-2, and might therefore pose an increased risk for future emergence from bats to humans (*17*).

Despite the risks associated with bat-origin coronaviruses, bats play integral roles in ecosystems, including insect suppression through predation, prey for numerous predators, pollinators for economically and ecologically useful plants, and seed dispersal for countless tropical trees and shrubs (*18*). Therefore, mitigating the risks of future emergence events from bats would benefit from minimizing close interaction between humans and bats and other wildlife, by reducing or stopping wildlife sales at wet markets, wildlife hunting, and encroachment on wildlife habitat.

Although further research on bats might help to understand the origins of coronaviruses, other wildlife species are intermediate hosts for human emerging coronaviruses. Intermediate hosts might not only add complexity to coronavirus transmission dynamics, but might also amplify viral spillover to new hosts by closing gaps in interaction frequency between species, and by increasing transmissibility and/or infectiousness through viral adaptation (*19*). A canonical example is SARS-CoV, whose intermediate host is accepted to be palm civets (Table 2; Appendix). In this instance, close interaction between humans and civets sold through wildlife markets probably facilitated transmission to humans, and passage and ongoing recombination in civet intermediate hosts is believed to have played a critical role in human receptor tropism (*19*,*20*) (Table 1).

Some wildlife species are at risk for human coronavirus spillover. Wild great apes, all species of which are endangered, are a taxonomic group vulnerable to spillover from humans, at least in part because they are our closest living relatives. Several documented respiratory outbreaks that resulted in clinical signs ranging from mild illness to death in chimpanzee and gorilla populations originated from a human source (*21*,*22*). The human betacoronavirus HCoV-OC43 was reported as the causative agent of mild-to-moderate respiratory illness among wild chimpanzees in Côte D’Ivoire in late 2016 and early 2017 (Table 2; Appendix), suggesting the susceptibility of these chimpanzees to human coronaviruses. As the COVID-19 pandemic continues, there is concern that susceptible wildlife, such as great apes, might be exposed to the virus through human contact, resulting in a new host reservoir, which could pose a risk for perpetuating enzootic transmission and zoonotic transmission into recovering human populations.

Wildlife infections with SARS-CoV-2 have already occurred; the first natural infection of SARS-CoV-2 in a wild animal, and the first confirmed animal cases in the United States, were in tigers (n = 5) and lions (n = 3) at a zoo in New York, NY (Table 2; Appendix). Unlike most other asymptomatic animal cases reported previously, the large cats demonstrated respiratory signs that included coughing and wheezing but ultimately made a full recovery (Table 3). SARS-CoV-2 infection in wild felids in captivity highlights the complex interactions humans might have with wildlife, including the potential for human-to-wildlife transmission. Given these interlinkages, framing risk by using a One Health approach might more comprehensively address the socioeconomic and environmental drivers of disease emergence, leading to potentially novel, mutually beneficial solutions. For example, risks could be reduced by improving wildlife importation, trade and market regulations, and sanitary standards, which would not only protect public health and animal health but also result in positive wildlife conservation outcomes.

## Emerging Coronaviruses and Livestock

Some coronaviruses naturally infect livestock and can have devastating economic consequences, such as swine acute diarrhea syndrome coronavirus (SADS-CoV), porcine epidemic diarrhea virus (PEDV), and betacoronavirus 1. Although recent studies suggest that pigs are not susceptible hosts for SARS-CoV-2 infection (*23*,*24*), pigs are a common host for alphacoronaviruses and betacoronaviruses; 6 viral species cause disease (*25*) (Table 2). Of these species, the enteric alphacoronavirus PEDV is considered reemerging, and the enteric alphacoronavirus SADS-CoV (a strain of the *Rhinolophus* bat coronavirus HKU2) is considered emerging (*25*). Although PEDV was detected in China in the 1970s, a highly pathogenic variant caused considerable losses to the United States pork industry in 2013–2014 (*26*). SADS-CoV is highly pathogenic in swine and was detected in Guangdong Province in China during 2016–2017, causing the death of nearly 25,000 piglets (*27*) (Table 3). SADS-CoV emerged within 100 km of the accepted locale of the SARS index case, and like SARS-CoV and SARS-CoV-2, SADS-CoV is suspected to originate in horseshoe bats (*Rhinolophus* spp.) (Table 2; Appendix). However, unlike SARS-CoV and SARS-CoV-2, SADS-CoV has not been detected outside China (*25*).

Among betacoronaviruses, a strain of betacoronavirus 1 also infects pigs (*25*). Porcine hemagglutinating encephalomyelitis virus has been circulating for decades and causes rapid death in piglets (*25*) (Table 3). Unlike other coronaviruses, betacoronavirus 1 is a unique species complex, in that its distinct strains are host-specific to a range of different species, including wild and domestic ungulates, rabbits, and canines (*19*,*28*) (Table 2; Appendix). Perhaps the most well-studied strain of betacoronavirus 1 is bovine coronavirus (BCoV), which has a major economic role because it can be associated with a suite of clinical disease in calves and cattle, including calf diarrhea, winter dysentery, and respiratory infection (*28*) (Table 3). BCoV also infects several other livestock species, including horses, sheep, and camels (*19*,*28*) (Table 2).

Livestock have also been intermediate hosts in the emergence of 3 human coronaviruses. An unknown ungulate species, speculated to be cattle, is accepted as the intermediate host of HCoV-OC43 (*6*,*29*), a strain of betacoronavirus 1 (Table 2). On the basis of molecular clock calculations, HCoV-OC43 is predicted to have jumped from livestock to humans around 1890, a timeframe coincident with pandemics of respiratory disease in cattle (which resulted in widespread culling) and humans (although this outbreak is historically attributed to influenza) (*6*). Dromedary camels are accepted as established hosts of MERS-CoV and are believed to be associated with the emergence of HCoV-229E in humans on the basis of closely related viruses found in camelids (Table 2; Appendix). Dromedary camels inhabit the Middle East and northern Africa and comprise 90% of extant camels on earth. In much of their range, dromedaries are a major livestock species that are used as racing and working animals, as well as for their milk, meat, and hides.

Livestock can also be spillover hosts of human coronavirus infection. After the 2002–2003 SARS outbreak, a study conducted on farms in Xiqing County, China, tested livestock (pigs, cattle, chickens, and ducks) and companion animals (dogs and cats), leading to detection of 1 pig that was positive for SARS-CoV by antibody test and reverse transcription PCR (*30*) (Table 2). A larger and more complex series of livestock outbreaks of SARS-CoV-2 has been unfolding since April 2020. Mink farms across Europe and North America have reported outbreaks of SARS-CoV-2 (Tables 2, 3). In most outbreaks, farmed mink were suspected to be initially infected by COVID-19–positive farm employees (*31*,*32*). Findings from the Netherlands have also identified instances of spillback from mink to humans through ongoing investigations (*33*). National surveillance and control efforts have been implemented in several countries, many of which have subsequently identified other SARS-CoV-2–positive species living on or nearby mink farms, including cats, dogs, and escaped or wild mink (*32*). Several countries have implemented mandatory reporting of any virus-positive animals and depopulation or quarantine of affected farms (*32*). In Europe, several million mink have been culled, and a moratorium has been placed on the mink industry in some countries; such early and coordinated One Health actions are needed to prevent bidirectional transmission of zoonotic diseases (*32*).

## Emerging Coronaviruses and Companion Animals

Companion animals are members of many households and can improve the physical and mental well-being of their owners (*34*). In the United States, ≈71.5 million households (57%) own >1 companion animal (*35*). Among households with companion animals, dogs (67%) and cats (44%) are the most commonly owned (*35*). Despite the many benefits of pet ownership, close interactions with pets pose risks for zoonotic disease transmission (*34*). Zoonotic diseases that are spread between humans and companion animals include rabies, salmonellosis, campylobacteriosis, and hookworm (*34*,*36*,*37*). Companion animals are estimated to be a source of >70 human diseases (*38*), and the burden of zoonotic diseases attributed to interactions with companion animals is substantial. For example, rabies kills ≈59,000 persons per year globally, and 99% of human rabies cases originate from rabid dogs (*37*).

Several common coronaviruses have been detected in companion animals, although none of the coronaviruses that are endemic to companion animal populations are zoonotic. One of the most common respiratory diseases in dogs is canine infectious respiratory disease, or kennel cough, which typically causes cough and nasal discharge in puppies and dogs (*39*,*40*). Although kennel cough can be caused by several pathogens, most frequently the bacterium *Bordetella bronchiseptica*, canine respiratory coronavirus (CRCoV) is a contributing pathogen to this syndrome (*39*,*41*) (Table 1). CRCoV is believed to originate from BCoV through a common ancestor, host variant, or a host species shift and is therefore considered a strain of betacoronavirus 1 (*39*,*41*). Regardless of how CRCoV and BCoV are genetically related, experimental studies have shown that dogs challenged with BCoV can become infected and transmit the virus to other dogs, although they do not exhibit clinical signs of disease (Tables 2, 3; Appendix).

Canine enteric coronavirus (CCoV) is an alphacoronavirus often associated with mild enteritis in puppies and dogs, especially in group housing situations (*42*). However, during 2005, a novel, highly pathogenic variant strain of CCoV-II, CB/05, was identified (*43*) (Table 2). This new variant is now pantropic, and results in a mortality rate up to 100% in isolated outbreaks in puppies (*43*) (Table 3). Because of its increased pathogenicity and changes in tissue tropism, CCoV is considered an emerging pathogen (*42*).

Although CCoV is generally considered to be specific to dogs, cats experimentally challenged with the virus can be infected with CCoV and mount an anamnestic response to further exposure, although they do not develop clinical signs of illness (Table 3; Appendix). In addition, although there are 2 serotypes of feline coronavirus (FCoV), FCoV type I and FCoV type II, type II is hypothesized to have originated from a recombination event between FCoV type I and CCoV, which suggests co-infections of coronaviruses among companion animals might yield opportunity for emergence of new disease (*44*).

Companion animals might also act as spillover hosts for human coronaviruses. A study after the 2002–2003 SARS outbreak showed that pet cats living in a Hong Kong, China, apartment complex were naturally infected with SARS during the epidemic (*45*). After the epidemic, challenge experiments in cats and ferrets found that both species could be experimentally infected and transmit the infection to immunologically naive animals of the same species they were housed with (*45*) (Table 2). In this experiment, cats did not show clinical signs of illness, although ferrets became lethargic, showed development of conjunctivitis, and died on days 16 and 21 postinfection. However, unlike human cases, there was no evidence that SARS-CoV–associated pneumonia was a cause of death (Table 3). Rather, the main findings in deceased ferrets were marked hepatic lipidosis and emaciation (*45*).

Companion animals, specifically dogs and cats, are among the most commonly infected groups of animals in the ongoing COVID-19 pandemic. Natural cases of suspected human-to-animal transmission have been confirmed in dogs and cats from several countries, and the earliest reports date back to March 2020 in Hong Kong (*32*). As of January 2021, there are ≈100 confirmed cases of SARS-CoV-2 infections in dogs and cats in the United States; most of those cases resulted from exposure to owners who had COVID-19 (*46*). Experimental challenge studies additionally suggest that similar to SARS-CoV, several companion animals, including cats, ferrets, and golden hamsters, are all susceptible to SARS-CoV-2 infection under laboratory conditions (Table 2; Appendix). Furthermore, studies in cats, hamsters, and ferrets showed that they are capable of direct and indirect transmission to healthy animals of the same species in experimental settings (*23*,*24*,*47*,*48*), which underscores the need for infection prevention and control practices for humans and companion animals (*49*).

The global prevalence of companion animal ownership underscores the need for better understanding of pathogens, such as coronaviruses, that can infect pets. Because companion animals harbor endemic coronaviruses and might also be at risk for spillover for some human zoonotic coronaviruses, there is potential for coronavirus recombination events and new viral emergence to occur within these hosts. Therefore, ensuring that persons understand how to safely interact with their companion animals is essential for ensuring that persons and companion animals stay healthy while also protecting animal welfare.

## Conclusions

A considerable number of mammalian species, including wildlife, livestock, and companion animals, are susceptible to infection with alphacoronaviruses and betacoronaviruses. The propensity of alphacoronaviruses and betacoronaviruses to jump to new hosts, coupled with their relatively large host ranges, suggests that a One Health approach could be used to develop strategies to mitigate the effects of current and future coronavirus emergence events. During the COVID-19 pandemic, One Health collaboration between public health and veterinary sectors has already bolstered critical healthcare resources and infrastructure, leading to improvements in diagnostic testing capacity and human resource availability (*50*). In the United States, the One Health Federal Interagency COVID-19 Coordination Group has developed risk communication and messaging for companion animals, livestock, and wildlife and has been instrumental in coordinating joint outbreak response and diagnostic testing in animals. As these examples highlight, integration of the One Health approach into preparedness planning, joint epidemiologic investigations, surveillance, laboratory diagnostics, risk assessment, and field research is not only beneficial but a useful approach to safeguard the health, welfare and safety of humans, animals, and their shared environment.

Appendix Additional information on animal reservoirs and hosts for emerging alphacoronaviruses and betacoronaviruses.
