# Supplementary material for: Animal Reservoirs and Hosts for Emerging Alphacoronaviruses and Betacoronaviruses
Source: Emerg Infect Dis. 2021 Apr;27(4):1015–22. doi: 10.3201/eid2704.203945 (PMC8007319; doi:10.3201/eid2704.203945)
Supplement: Appendix — Additional information on animal reservoirs and hosts for emerging alphacoronaviruses and betacoronaviruses. [file 20-3945-Techapp-s1.pdf]

Article DOI: https://doi.org/10.3201/eid2704.203945

Animal Reservoirs and Hosts for Emerging Alphacoronaviruses and Betacoronaviruses

Appendix

Appendix Table. Citations for in-text tables, by coronavirus and host category

Pathogen (abbreviation)	Category	Table	Reference
Alphacoronavirus 1 (ACoV1); strain canine enteric coronavirus (CCoV)	Receptor	1	(*1*)
	Reservoir host(s)	2	(*2*)
	Spillover host(s)	2	(*3–6*)
	Clinical manifestation	3	(*3–9*)
Alphacoronavirus 1 (ACoV1); strain feline infectious peritonitis virus (FIPV)	Receptor	1	(*10*)
	Reservoir host(s)	2	(*11,12*)
	Spillover host(s)	2	(*13–15*)
	Susceptible host	2	(*16*)
	Clinical manifestation	3	(*7,9,17,18*)
Bat coronavirus HKU10	Receptor	1	(*19*)
	Reservoir host(s)	2	(*20*)
	Spillover host(s)	2	(*21*)
	Clinical manifestation	3	(*9,21*)
Ferret systemic coronavirus (FRSCV)	Receptor	1	(*22*)
	Reservoir host(s)	2	(*23*)
	Spillover host(s)	2	(*24,25*)
	Clinical manifestation	3	(*9,26*)
Human coronavirus NL63	Receptor	1	(*27*)
	Reservoir host(s)	2	(*28*)
	Spillover host(s)	2	(*29,30*)
	Nonsusceptible host(s)	2	(*31*)
	Clinical manifestation	3	(*9,32–34*)
Human coronavirus 229E	Receptor	1	(*35*)
	Reservoir host(s)	2	(*28,36,37*)
	Intermediate host(s)	2	(*38*)
	Spillover host(s)	2	(*39,40*)
	Susceptible host(s)	2	(*41*)
	Clinical manifestation	3	(*9,32,34,38,41–43*)
Porcine epidemic diarrhea virus (PEDV)	Receptor	1	(*44,45*)
	Reservoir host(s)	2	(*32,46*)
	Spillover host(s)	2	(*47*)
	Clinical manifestation	3	(*7,9,32,48*)
Rhinolophus bat coronavirus HKU2; strain swine acute diarrhea syndrome coronavirus (SADS-CoV)	Receptor	1	(*49*)
	Reservoir host(s)	2	(*49*)
	Spillover host(s)	2	(*49*)
	Susceptible host(s)	2	(*50*)
	Clinical manifestation	3	(*9,32*)
Betacoronavirus 1 (BCoV1); strain bovine coronavirus	Receptor	1	(*51*)
	Reservoir host(s)	2	(*52*)
	Spillover host(s)	2	(*53*)
	Susceptible host(s)	2	(*54*)
	Nonsusceptible host(s)	2	(*54*)
	Clinical manifestation	3	(*7,9,52,54*)
Betacoronavirus 1 (BCoV1); strain canine respiratory coronavirus	Receptor	1	(*51*)
	Reservoir host(s)	2	(*52*)
	Intermediate host(s)	2	(*55*)
	Spillover host(s)	2	(*56*)
	Clinical manifestation	3	(*7,9,52,56,57*)
Betacoronavirus1 (BCoV1); strain human coronavirus OC43	Receptor	1	(*58*)
	Reservoir host(s)	2	(*52*)
	Intermediate host(s)	2	(*59*)
	Spillover host(s)	2	(*60,61*)
	Susceptible host(s)	2	(*62*)
	Clinical manifestation	3	(*9,34,52,57,61–63*)
Human coronavirus HKU1	Receptor	1	(*51,64*)
	Reservoir host(s)	2	(*65,66*)
	Spillover host(s)	2	(*67*)

Pathogen (abbreviation)	Category	Table	Reference
	Clinical manifestation	3	(*9,34,66,68*)
Middle East respiratory syndrome coronavirus (MERS-CoV)	Receptor	1	(*69*)
	Reservoir host(s)	2	(*65,70*)
	Intermediate host(s)	2	(*71*)
	Spillover host(s)	2	(*72*)
	Susceptible host(s)	2	(*73–79*)
	Nonsusceptible host(s)	2	(*76,80–82*)
	Clinical manifestation	3	(*9,32,73,74,76,79,83,84*)
Severe acute respiratory syndrome coronavirus (SARS-CoV)	Receptor	1	(*85*)
	Reservoir host(s)	2	(*19,86–88*)
	Intermediate host(s)	2	(*89,90*)
	Spillover host(s)	2	(*87,90–95*)
	Susceptible host(s)	2	(*87,91,96–101*)
	Nonsusceptible host(s)	2	(*97*)
	Clinical manifestation	3	(*9,17,32,84,87,91,92,96–101*)
Severe acute respiratory syndrome coronavirus 2 (SARS-CoV-2)	Receptor	1	(*102*)
	Reservoir host(s)	2	(*103*)
	Spillover host(s)	2	(*104–109*)
	Susceptible host(s)	2	(*110–125*)
	Nonsusceptible host(s)	2	(*111,113,126–129*)
	Clinical manifestation	3	(*103–106,108,110,111,113–119,121,122,124,130*)

References

1. Licitra BN, Duhamel GE, Whittaker GR. Canine enteric coronaviruses: emerging viral pathogens with distinct recombinant spike proteins. Viruses. 2014;6:3363–76. PubMed https://doi.org/10.3390/v6083363

2. Binn LN, Lazar EC, Keenan KP, Huxsoll DL, Marchwicki RH, Strano AJ. Recovery and characterization of a coronavirus from military dogs with diarrhea. Proc Annu Meet US Anim Health Assoc. 1974;78:359–66. PubMed

3. Wang Y, Ma G, Lu C, Wen H. Detection of canine coronaviruses genotype I and II in raised Canidae animals in China. Berl Munch Tierarztl Wochenschr. 2006;119:35–9. PubMed

4. Rosa GM, Santos N, Grøndahl-Rosado R, Fonseca FP, Tavares L, Neto I, et al. Unveiling patterns of viral pathogen infection in free-ranging carnivores of northern Portugal using a complementary methodological approach. Comp Immunol Microbiol Infect Dis. 2020;69:101432. PubMed https://doi.org/10.1016/j.cimid.2020.101432

5. Zarnke RL, Evermann J, Ver Hoef JM, McNay ME, Boertje RD, Gardner CL, et al. Serologic survey for canine coronavirus in wolves from Alaska. J Wildl Dis. 2001;37:740–5. PubMed https://doi.org/10.7589/0090-3558-37.4.740

6. McArdle F, Bennett M, Gaskell RM, Tennant B, Kelly DF, Gaskell CJ. Induction and enhancement of feline infectious peritonitis by canine coronavirus. Am J Vet Res. 1992;53:1500–6. PubMed

7. American Veterinary Medical Association. Coronavirus in domestic species. Schaumburg (IL): The Association; 2020.

8. Pratelli A. Genetic evolution of canine coronavirus and recent advances in prophylaxis. Vet Res. 2006;37:191–200. PubMed https://doi.org/10.1051/vetres:2005053

9. US Department of Agriculture. Veterinary biological products. Ames (IA): The Department; 2020.

10. Hohdatsu T, Izumiya Y, Yokoyama Y, Kida K, Koyama H. Differences in virus receptor for type I and type II feline infectious peritonitis virus. Arch Virol. 1998;143:839–50. PubMed https://doi.org/10.1007/s007050050336

11. Poland AM, Vennema H, Foley JE, Pedersen NC. Two related strains of feline infectious peritonitis virus isolated from immunocompromised cats infected with a feline enteric coronavirus. J Clin Microbiol. 1996;34:3180–4. PubMed https://doi.org/10.1128/JCM.34.12.3180-3184.1996

12. Vennema H, Poland A, Foley J, Pedersen NC. Feline infectious peritonitis viruses arise by mutation from endemic feline enteric coronaviruses. Virology. 1998;243:150–7. PubMed https://doi.org/10.1006/viro.1998.9045

13. Evermann JF, Heeney JL, Roelke ME, McKeirnan AJ, O'Brien SJ. Biological and pathological consequences of feline infectious peritonitis virus infection in the cheetah. Arch Virol. 1988;102:155–71. PubMed https://doi.org/10.1007/BF01310822

14. Mwase M, Shimada K, Mumba C, Yabe J, Squarre D, Madarame H. Positive immunolabelling for feline infectious peritonitis in an African lion (*Panthera leo*) with bilateral panuveitis. J Comp Pathol. 2015;152:265–8. PubMed https://doi.org/10.1016/j.jcpa.2014.12.006

15. Foley JE, Swift P, Fleer KA, Torres S, Girard YA, Johnson CK. Risk factors for exposure to feline pathogens in California mountain lions (*Puma concolor*). J Wildl Dis. 2013;49:279–93. PubMed https://doi.org/10.7589/2012-08-206

16. Horzinek MC, Osterhaus AD, Wirahadiredja RM, de Kreek P. Feline infectious peritonitis (FIP) virus. III. Studies on the multiplication of FIP virus in the suckling mouse. Zentralbl Veterinärmed B. 1978;25:806–15. PubMed https://doi.org/10.1111/j.1439-0450.1978.tb01056.x

17. Levy JK. Overview of feline infectious peritonitis. Merck Veterinary Manual; 2014 [cited 2021 Jan 22]. https://www.merckvetmanual.com/generalized-conditions/feline-infectious-peritonitis/overview-of-feline-infectious-peritonitis

18. Pedersen NC. An update on feline infectious peritonitis: diagnostics and therapeutics. Vet J. 2014;201:133–41. PubMed https://doi.org/10.1016/j.tvjl.2014.04.016

19. Lau SK, Woo PC, Li KS, Huang Y, Tsoi H-W, Wong BH, et al. Severe acute respiratory syndrome coronavirus-like virus in Chinese horseshoe bats. Proc Natl Acad Sci U S A. 2005;102:14040–5. PubMed https://doi.org/10.1073/pnas.0506735102

20. Woo PC, Wang M, Lau SK, Xu H, Poon RW, Guo R, et al. Comparative analysis of twelve genomes of three novel group 2c and group 2d coronaviruses reveals unique group and subgroup features. J Virol. 2007;81:1574–85. PubMed https://doi.org/10.1128/JVI.02182-06

21. Lau SK, Li KS, Tsang AK, Shek C-T, Wang M, Choi GK, et al. Recent transmission of a novel alphacoronavirus, bat coronavirus HKU10, from Leschenault's rousettes to pomona leaf-nosed bats: first evidence of interspecies transmission of coronavirus between bats of different suborders. J Virol. 2012;86:11906–18. PubMed https://doi.org/10.1128/JVI.01305-12

22. Murray J, Kiupel M, Maes RK. Ferret coronavirus-associated diseases. Vet Clin North Am Exot Anim Pract. 2010;13:543–60. PubMed https://doi.org/10.1016/j.cvex.2010.05.010

23. Wise AG, Kiupel M, Maes RK. Molecular characterization of a novel coronavirus associated with epizootic catarrhal enteritis (ECE) in ferrets. Virology. 2006;349:164–74. PubMed https://doi.org/10.1016/j.virol.2006.01.031

24. Garner MM, Ramsell K, Morera N, Juan-Sallés C, Jiménez J, Ardiaca M, et al. Clinicopathologic features of a systemic coronavirus-associated disease resembling feline infectious peritonitis in the domestic ferret (*Mustela putorius*). Vet Pathol. 2008;45:236–46. PubMed https://doi.org/10.1354/vp.45-2-236

25. Xu Y. Genetic diversity and potential recombination between ferret coronaviruses from European and American lineages. J Infect. 2020;80:350–71. PubMed https://doi.org/10.1016/j.jinf.2020.01.016

26. Morrisey JK. Infectious diseases of Ferrets. Merck Veterinary Manual; 2013 [cited 2021 Jan 22]. https://www.merckvetmanual.com/exotic-and-laboratory-animals/ferrets/infectious-diseases-of-ferrets

27. Hofmann H, Pyrc K, van der Hoek L, Geier M, Berkhout B, Pöhlmann S. Human coronavirus NL63 employs the severe acute respiratory syndrome coronavirus receptor for cellular entry. Proc Natl Acad Sci U S A. 2005;102:7988–93. PubMed https://doi.org/10.1073/pnas.0409465102

28. Tao Y, Shi M, Chommanard C, Queen K, Zhang J, Markotter W, et al. Surveillance of bat coronaviruses in Kenya identifies relatives of human coronaviruses NL63 and 229E and their recombination history. J Virol. 2017;91:e01953–16. PubMed https://doi.org/10.1128/JVI.01953-16

29. van der Hoek L, Pyrc K, Jebbink MF, Vermeulen-Oost W, Berkhout RJ, Wolthers KC, et al. Identification of a new human coronavirus. Nat Med. 2004;10:368–73. PubMed https://doi.org/10.1038/nm1024

30. Fouchier RA, Hartwig NG, Bestebroer TM, Niemeyer B, de Jong JC, Simon JH, et al. A previously undescribed coronavirus associated with respiratory disease in humans. Proc Natl Acad Sci U S A. 2004;101:6212–6. PubMed https://doi.org/10.1073/pnas.0400762101

31. El-Duah P, Meyer B, Sylverken A, Owusu M, Gottula LT, Yeboah R, et al. Development of a whole-virus ELISA for serological evaluation of domestic livestock as possible hosts of human coronavirus NL63. Viruses. 2019;11:43. PubMed https://doi.org/10.3390/v11010043

32. Banerjee A, Kulcsar K, Misra V, Frieman M, Mossman K. Bats and coronaviruses. Viruses. 2019;11:41. PubMed https://doi.org/10.3390/v11010041

33. Abdul-Rasool S, Fielding BC. Understanding human coronavirus HCoV-NL63. Open Virol J. 2010;4:76–84. PubMed https://doi.org/10.2174/1874357901004010076

34. Center for Disease Control and Prevention. Common human coronaviruses. Coronavirus 2020 [cited 2020 Jul 13]. https://www.cdc.gov/coronavirus/general-information.html

35. Yeager CL, Ashmun RA, Williams RK, Cardellichio CB, Shapiro LH, Look AT, et al. Human aminopeptidase N is a receptor for human coronavirus 229E. Nature. 1992;357:420–2. PubMed https://doi.org/10.1038/357420a0

36. Pfefferle S, Oppong S, Drexler JF, Gloza-Rausch F, Ipsen A, Seebens A, et al. Distant relatives of severe acute respiratory syndrome coronavirus and close relatives of human coronavirus 229E in bats, Ghana. Emerg Infect Dis. 2009;15:1377–84. PubMed https://doi.org/10.3201/eid1509.090224

37. Corman VM, Baldwin HJ, Tateno AF, Zerbinati RM, Annan A, Owusu M, et al. Evidence for an ancestral association of human coronavirus 229E with bats. J Virol. 2015;89:11858–70. PubMed https://doi.org/10.1128/JVI.01755-15

38. Corman VM, Eckerle I, Memish ZA, Liljander AM, Dijkman R, Jonsdottir H, et al. Link of a ubiquitous human coronavirus to dromedary camels. Proc Natl Acad Sci U S A. 2016;113:9864–9. PubMed https://doi.org/10.1073/pnas.1604472113

39. Crossley BM, Mock RE, Callison SA, Hietala SK. Identification and characterization of a novel alpaca respiratory coronavirus most closely related to the human coronavirus 229E. Viruses. 2012;4:3689–700. PubMed https://doi.org/10.3390/v4123689

40. Hamre D, Procknow JJ. A new virus isolated from the human respiratory tract. Proc Soc Exp Biol Med. 1966;121:190–3. PubMed https://doi.org/10.3181/00379727-121-30734

41. Barlough JE, Johnson-Lussenburg CM, Stoddart CA, Jacobson RH, Scott FW. Experimental inoculation of cats with human coronavirus 229E and subsequent challenge with feline infectious peritonitis virus. Can J Comp Med. 1985;49:303–7. PubMed

42. Crossley BM, Barr BC, Magdesian KG, Ing M, Mora D, Jensen D, et al. Identification of a novel coronavirus possibly associated with acute respiratory syndrome in alpacas (*Vicugna pacos*) in California, 2007. J Vet Diagn Invest. 2010;22:94–7. PubMed https://doi.org/10.1177/104063871002200118

43. McIntosh K. Coronaviruses [cited 2020 Feb 18]. https://www.uptodate.com/contents/coronaviruses

44. Liu C, Tang J, Ma Y, Liang X, Yang Y, Peng G, et al. Receptor usage and cell entry of porcine epidemic diarrhea coronavirus. J Virol. 2015;89:6121–5. PubMed https://doi.org/10.1128/JVI.00430-15

45. Shirato K, Maejima M, Islam MT, Miyazaki A, Kawase M, Matsuyama S, et al. Porcine aminopeptidase N is not a cellular receptor of porcine epidemic diarrhea virus, but promotes its infectivity via aminopeptidase activity. J Gen Virol. 2016;97:2528–39. PubMed https://doi.org/10.1099/jgv.0.000563

46. Tang XC, Zhang JX, Zhang SY, Wang P, Fan XH, Li LF, et al. Prevalence and genetic diversity of coronaviruses in bats from China. J Virol. 2006;80:7481–90. PubMed https://doi.org/10.1128/JVI.00697-06

47. Lee C. Porcine epidemic diarrhea virus: An emerging and re-emerging epizootic swine virus. Virol J. 2015;12:193. PubMed https://doi.org/10.1186/s12985-015-0421-2

48. Song D, Moon H, Kang B. Porcine epidemic diarrhea: a review of current epidemiology and available vaccines. Clin Exp Vaccine Res. 2015;4:166–76. PubMed https://doi.org/10.7774/cevr.2015.4.2.166

49. Zhou P, Fan H, Lan T, Yang X-L, Shi W-F, Zhang W, et al. Fatal swine acute diarrhoea syndrome caused by an HKU2-related coronavirus of bat origin. Nature. 2018;556:255–8. PubMed https://doi.org/10.1038/s41586-018-0010-9

50. Yang Y-L, Qin P, Wang B, Liu Y, Xu G-H, Peng L, et al. Broad cross-species infection of cultured cells by bat HKU2-related swine acute diarrhea syndrome coronavirus and identification of its replication in murine dendritic cells in vivo highlight its potential for diverse interspecies transmission. J Virol. 2019;93:e01448–19. PubMed https://doi.org/10.1128/JVI.01448-19

51. Szczepanski A, Owczarek K, Bzowska M, Gula K, Drebot I, Ochman M, et al. Canine respiratory coronavirus, bovine coronavirus, and human coronavirus OC43: receptors and attachment factors. Viruses. 2019;11:328. PubMed https://doi.org/10.3390/v11040328

52. Lau SK, Woo PC, Li KS, Tsang AK, Fan RY, Luk HK, et al. Discovery of a novel coronavirus, China *Rattus* coronavirus HKU24, from Norway rats supports the murine origin of betacoronavirus 1 and has implications for the ancestor of betacoronavirus lineage A. J Virol. 2015;89:3076–92. PubMed https://doi.org/10.1128/JVI.02420-14

53. Storz J, Stine L, Liem A, Anderson GA. Coronavirus isolation from nasal swab samples in cattle with signs of respiratory tract disease after shipping. J Am Vet Med Assoc. 1996;208:1452–5. PubMed

54. Ismail MM, Cho KO, Ward LA, Saif LJ, Saif YM. Experimental bovine coronavirus in turkey poults and young chickens. Avian Dis. 2001;45:157–63. PubMed https://doi.org/10.2307/1593023

55. Erles K, Shiu K-B, Brownlie J. Isolation and sequence analysis of canine respiratory coronavirus. Virus Res. 2007;124:78–87. PubMed https://doi.org/10.1016/j.virusres.2006.10.004

56. Erles K, Toomey C, Brooks HW, Brownlie J. Detection of a group 2 coronavirus in dogs with canine infectious respiratory disease. Virology. 2003;310:216–23. PubMed https://doi.org/10.1016/S0042-6822(03)00160-0

57. Erles K, Brownlie J. Canine respiratory coronavirus: an emerging pathogen in the canine infectious respiratory disease complex. Vet Clin North Am Small Anim Pract. 2008;38:815–25, viii. PubMed https://doi.org/10.1016/j.cvsm.2008.02.008

58. Collins AR. HLA class I antigen serves as a receptor for human coronavirus OC43. Immunol Invest. 1993;22:95–103. PubMed https://doi.org/10.3109/08820139309063393

59. Vijgen L, Keyaerts E, Moës E, Thoelen I, Wollants E, Lemey P, et al. Complete genomic sequence of human coronavirus OC43: molecular clock analysis suggests a relatively recent zoonotic coronavirus transmission event. J Virol. 2005;79:1595–604. PubMed https://doi.org/10.1128/JVI.79.3.1595-1604.2005

60. Patrono LV, Samuni L, Corman VM, Nourifar L, Röthemeier C, Wittig RM, et al. Human coronavirus OC43 outbreak in wild chimpanzees, Côte d´Ivoire, 2016. Emerg Microbes Infect. 2018;7:118. PubMed https://doi.org/10.1038/s41426-018-0121-2

61. McIntosh K, Dees JH, Becker WB, Kapikian AZ, Chanock RM. Recovery in tracheal organ cultures of novel viruses from patients with respiratory disease. Proc Natl Acad Sci U S A. 1967;57:933–40. PubMed https://doi.org/10.1073/pnas.57.4.933

62. Jacomy H, Talbot PJ. Vacuolating encephalitis in mice infected by human coronavirus OC43. Virology. 2003;315:20–33. PubMed https://doi.org/10.1016/S0042-6822(03)00323-4

63. Patrono LV, Samuni L, Corman VM, Nourifar L, Röthemeier C, Wittig RM, et al. Human coronavirus OC43 outbreak in wild chimpanzees, Côte d'Ivoire, 2016. Emerg Microbes Infect. 2018;7:118. PubMed https://doi.org/10.1038/s41426-018-0121-2

64. Chan CM, Lau SK, Woo PC, Tse H, Zheng B-J, Chen L, et al. Identification of major histocompatibility complex class I C molecule as an attachment factor that facilitates coronavirus HKU1 spike-mediated infection. J Virol. 2009;83:1026–35. PubMed https://doi.org/10.1128/JVI.01387-08

65. Wang W, Lin X-D, Guo W-P, Zhou R-H, Wang M-R, Wang C-Q, et al. Discovery, diversity and evolution of novel coronaviruses sampled from rodents in China. Virology. 2015;474:19–27. PubMed https://doi.org/10.1016/j.virol.2014.10.017

66. Corman VM, Muth D, Niemeyer D, Drosten C. Hosts and sources of endemic human coronaviruses. In: Kielian M, Mettenleiter TC, Roossinck MJ, editors. Advances in Virus Research. New York: Academic Press; 2018. p. 163–88.

67. Woo PC, Lau SK, Chu CM, Chan KH, Tsoi HW, Huang Y, et al. Characterization and complete genome sequence of a novel coronavirus, coronavirus HKU1, from patients with pneumonia. J Virol. 2005;79:884–95. PubMed https://doi.org/10.1128/JVI.79.2.884-895.2005

68. Esper F, Weibel C, Ferguson D, Landry ML, Kahn JS. Coronavirus HKU1 infection in the United States. Emerg Infect Dis. 2006;12:775–9. PubMed https://doi.org/10.3201/eid1205.051316

69. Meyerholz DK, Lambertz AM, McCray PB Jr. Dipeptidyl peptidase 4 distribution in the human respiratory tract: implications for the Middle East respiratory syndrome. Am J Pathol. 2016;186:78–86. PubMed https://doi.org/10.1016/j.ajpath.2015.09.014

70. Anthony SJ, Gilardi K, Menachery VD, Goldstein T, Ssebide B, Mbabazi R, et al. Further evidence for bats as the evolutionary source of Middle East respiratory syndrome coronavirus. MBio. 2017;8:e00373–17. PubMed https://doi.org/10.1128/mBio.00373-17

71. Reusken CB, Raj VS, Koopmans MP, Haagmans BL. Cross host transmission in the emergence of MERS coronavirus. Curr Opin Virol. 2016;16:55–62. PubMed https://doi.org/10.1016/j.coviro.2016.01.004

72. Zaki AM, van Boheemen S, Bestebroer TM, Osterhaus AD, Fouchier RA. Isolation of a novel coronavirus from a man with pneumonia in Saudi Arabia. N Engl J Med. 2012;367:1814–20. PubMed https://doi.org/10.1056/NEJMoa1211721

73. Adney DR, Bielefeldt-Ohmann H, Hartwig AE, Bowen RA. Infection, replication, and transmission of Middle East respiratory syndrome coronavirus in alpacas. Emerg Infect Dis. 2016;22:1031–7. PubMed https://doi.org/10.3201/eid2206.160192

74. Falzarano D, de Wit E, Feldmann F, Rasmussen AL, Okumura A, Peng X, et al. Infection with MERS-CoV causes lethal pneumonia in the common marmoset. PLoS Pathog. 2014;10:e1004250. PubMed https://doi.org/10.1371/journal.ppat.1004250

75. Johnson RF, Via LE, Kumar MR, Cornish JP, Yellayi S, Huzella L, et al. Intratracheal exposure of common marmosets to MERS-CoV Jordan-n3/2012 or MERS-CoV EMC/2012 isolates does not result in lethal disease. Virology. 2015;485:422–30. PubMed https://doi.org/10.1016/j.virol.2015.07.013

76. Vergara-Alert J, van den Brand JMA, Widagdo W, Muñoz M V, Raj S, Schipper D, et al. Livestock susceptibility to infection with Middle East respiratory syndrome coronavirus. Emerg Infect Dis. 2017;23:232–40. PubMed https://doi.org/10.3201/eid2302.161239

77. de Wit E, Rasmussen AL, Falzarano D, Bushmaker T, Feldmann F, Brining DL, et al. Middle East respiratory syndrome coronavirus (MERS-CoV) causes transient lower respiratory tract infection in rhesus macaques. Proc Natl Acad Sci U S A. 2013;110:16598–603. PubMed https://doi.org/10.1073/pnas.1310744110

78. Yao Y, Bao L, Deng W, Xu L, Li F, Lv Q, et al. An animal model of MERS produced by infection of rhesus macaques with MERS coronavirus. J Infect Dis. 2014;209:236–42. PubMed https://doi.org/10.1093/infdis/jit590

79. Haagmans BL, van den Brand JM, Provacia LB, Raj VS, Stittelaar KJ, Getu S, et al. Asymptomatic Middle East respiratory syndrome coronavirus infection in rabbits. J Virol. 2015;89:6131–5. PubMed https://doi.org/10.1128/JVI.00661-15

80. Raj VS, Smits SL, Provacia LB, van den Brand JM, Wiersma L, Ouwendijk WJ, et al. Adenosine deaminase acts as a natural antagonist for dipeptidyl peptidase 4-mediated entry of the Middle East respiratory syndrome coronavirus. J Virol. 2014;88:1834–8. PubMed https://doi.org/10.1128/JVI.02935-13

81. de Wit E, Prescott J, Baseler L, Bushmaker T, Thomas T, Lackemeyer MG, et al. The Middle East respiratory syndrome coronavirus (MERS-CoV) does not replicate in Syrian hamsters. PLoS One. 2013;8:e69127. PubMed https://doi.org/10.1371/journal.pone.0069127

82. Cockrell AS, Peck KM, Yount BL, Agnihothram SS, Scobey T, Curnes NR, et al. Mouse dipeptidyl peptidase 4 is not a functional receptor for Middle East respiratory syndrome coronavirus infection. J Virol. 2014;88:5195–9. PubMed https://doi.org/10.1128/JVI.03764-13

83. Baseler L, de Wit E, Feldmann H. A comparative review of animal models of Middle East respiratory syndrome coronavirus infection. Vet Pathol. 2016;53:521–31. PubMed https://doi.org/10.1177/0300985815620845

84. Precision Vaccinations. Coronavirus vaccines. 2020 [cited 2021 Jan 22]. https://www.precisionvaccinations.com/vaccines/coronavirus-vaccines

85. Li W, Moore MJ, Vasilieva N, Sui J, Wong SK, Berne MA, et al. Angiotensin-converting enzyme 2 is a functional receptor for the SARS coronavirus. Nature. 2003;426:450–4. PubMed https://doi.org/10.1038/nature02145

86. Li W, Shi Z, Yu M, Ren W, Smith C, Epstein JH, et al. Bats are natural reservoirs of SARS-like coronaviruses. Science. 2005;310:676–9. PubMed https://doi.org/10.1126/science.1118391

87. Wang L-F, Shi Z, Zhang S, Field H, Daszak P, Eaton BT. Review of bats and SARS. Emerg Infect Dis. 2006;12:1834–40. PubMed https://doi.org/10.3201/eid1212.060401

88. Hu B, Ge X, Wang L-F, Shi Z. Bat origin of human coronaviruses. Virol J. 2015;12:221. PubMed https://doi.org/10.1186/s12985-015-0422-1

89. Wang M, Yan M, Xu H, Liang W, Kan B, Zheng B, et al. SARS-CoV infection in a restaurant from palm civet. Emerg Infect Dis. 2005;11:1860–5. PubMed https://doi.org/10.3201/eid1112.041293

90. Guan Y, Zheng BJ, He YQ, Liu XL, Zhuang ZX, Cheung CL, et al. Isolation and characterization of viruses related to the SARS coronavirus from animals in southern China. Science. 2003;302:276–8. PubMed https://doi.org/10.1126/science.1087139

91. Martina BE, Haagmans BL, Kuiken T, Fouchier RA, Rimmelzwaan GF, Van Amerongen G, et al. Virology: SARS virus infection of cats and ferrets. Nature. 2003;425:915. PubMed https://doi.org/10.1038/425915a

92. World Health Organization. Consensus document on the epidemiology of severe acute respiratory syndrome (SARS). Geneva: The Organization; 2003.

93. Chen W, Yan M, Yang L, Ding B, He B, Wang Y, et al. SARS-associated coronavirus transmitted from human to pig. Emerg Infect Dis. 2005;11:446–8. PubMed https://doi.org/10.3201/eid1103.040824

94. Xu R-H, He J-F, Evans MR, Peng G-W, Field HE, Yu D-W, et al. Epidemiologic clues to SARS origin in China. Emerg Infect Dis. 2004;10:1030–7. PubMed https://doi.org/10.3201/eid1006.030852

95. Lee SH. The SARS epidemic in Hong Kong. J Epidemiol Community Health. 2003;57:652–4. PubMed https://doi.org/10.1136/jech.57.9.652

96. Fouchier RA, Kuiken T, Schutten M, van Amerongen G, van Doornum GJ, van den Hoogen BG, et al. Aetiology: Koch’s postulates fulfilled for SARS virus. Nature. 2003;423:240. PubMed https://doi.org/10.1038/423240a

97. Weingartl HM, Copps J, Drebot MA, Marszal P, Smith G, Gren J, et al. Susceptibility of pigs and chickens to SARS coronavirus. Emerg Infect Dis. 2004;10:179–84. PubMed https://doi.org/10.3201/eid1002.030677

98. Roberts A, Vogel L, Guarner J, Hayes N, Murphy B, Zaki S, et al. Severe acute respiratory syndrome coronavirus infection of golden Syrian hamsters. J Virol. 2005;79:503–11. PubMed https://doi.org/10.1128/JVI.79.1.503-511.2005

99. Wu D, Tu C, Xin C, Xuan H, Meng Q, Liu Y, et al. Civets are equally susceptible to experimental infection by two different severe acute respiratory syndrome coronavirus isolates. J Virol. 2005;79:2620–5. PubMed https://doi.org/10.1128/JVI.79.4.2620-2625.2005

100. Roberts A, Paddock C, Vogel L, Butler E, Zaki S, Subbarao K. Aged BALB/c mice as a model for increased severity of severe acute respiratory syndrome in elderly humans. J Virol. 2005;79:5833–8. PubMed https://doi.org/10.1128/JVI.79.9.5833-5838.2005

101. Qin C, Wang J, Wei Q, She M, Marasco WA, Jiang H, et al. An animal model of SARS produced by infection of *Macaca mulatta* with SARS coronavirus. J Pathol. 2005;206:251–9. PubMed https://doi.org/10.1002/path.1769

102. Zhang H, Penninger JM, Li Y, Zhong N, Slutsky AS. Angiotensin-converting enzyme 2 (ACE2) as a SARS-CoV-2 receptor: molecular mechanisms and potential therapeutic target. Intensive Care Med. 2020;46:586–90. PubMed https://doi.org/10.1007/s00134-020-05985-9

103. Zhou P, Yang X-L, Wang X-G, Hu B, Zhang L, Zhang W, et al. A pneumonia outbreak associated with a new coronavirus of probable bat origin. Nature. 2020;579:270–3. PubMed https://doi.org/10.1038/s41586-020-2012-7

104. Newman A, Smith D, Ghai RR, Wallace RM, Torchetti MK, Loiacono C, et al. First reported cases of SARS-CoV-2 infection in companion animals—New York, March–April 2020. MMWR Morb Mortal Wkly Rep. 2020;69:710–3. PubMed https://doi.org/10.15585/mmwr.mm6923e3

105. World Organisation for Animal Health. COVID 19 portal events in Animals; 2020 [cited 2020 Jul 13]. : https://www.oie.int/en/scientific-expertise/specific-information-and-recommendations/questions-and-answers-on-2019novel-coronavirus/events-in-animals

106. Sit TH, Brackman CJ, Ip SM, Tam KW, Law PY, To E, et al. Infection of dogs with SARS-CoV-2. Nature. 2020;586:776–8. PubMed https://doi.org/10.1038/s41586-020-2334-5

107. Zhu N, Zhang D, Wang W, Li X, Yang B, Song J, et al.; China Novel Coronavirus Investigating and Research Team. A novel coronavirus from patients with pneumonia in China, 2019. N Engl J Med. 2020;382:727–33. PubMed https://doi.org/10.1056/NEJMoa2001017

108. Oreshkova N, Molenaar R-J, Vreman S, Harders F, Oude Munnink BB, Hakze-van der Honing RW, et al. SARS-CoV-2 infection in farmed minks, the Netherlands, April and May 2020. Euro Surveill. 2020;25:2001005. PubMed https://doi.org/10.2807/1560-7917.ES.2020.25.23.2001005

109. Molenaar RJ, Vreman S, Hakze-van der Honing RW, Zwart R, de Rond J, Weesendorp E, et al. Clinical and pathological findings in SARS-CoV-2 disease outbreaks in farmed mink. Vet Pathol. 2020;57:653–7. PubMed https://doi.org/10.1177/0300985820943535

110. Rockx B, Kuiken T, Herfst S, Bestebroer T, Lamers MM, Oude Munnink BB, et al. Comparative pathogenesis of COVID-19, MERS, and SARS in a nonhuman primate model. Science. 2020;368:1012–5. PubMed https://doi.org/10.1126/science.abb7314

111. Shi J, Wen Z, Zhong G, Yang H, Wang C, Huang B, et al. Susceptibility of ferrets, cats, dogs, and other domesticated animals to SARS-coronavirus 2. Science. 2020;368:1016–20. PubMed https://doi.org/10.1126/science.abb7015

112. Halfmann PJ, Hatta M, Chiba S, Maemura T, Fan S, Takeda M, et al. Transmission of SARS-CoV-2 in domestic cats. N Engl J Med. 2020;383:592–4. PubMed https://doi.org/10.1056/NEJMc2013400

113. Schlottau K, Rissmann M, Graaf A, Schön J, Sehl J, Wylezich C, et al. Experimental transmission studies of SARS-CoV-2 in fruit bats, ferrets, pigs and chickens. Lancet Microbe. 2020;1:e218–25. PubMed https://doi.org/10.1016/S2666-5247(20)30089-6

114. Kim Y-I, Kim S-G, Kim S-M, Kim E-H, Park S-J, Yu K-M, et al. Infection and rapid transmission of SARS-CoV-2 in ferrets. Cell Host Microbe. 2020;27:704–709.e2. PubMed https://doi.org/10.1016/j.chom.2020.03.023

115. Cross RW, Agans KN, Prasad AN, Borisevich V, Woolsey C, Deer DJ, et al. Intranasal exposure of African green monkeys to SARS-CoV-2 results in acute phase pneumonia with shedding and lung injury still present in the early convalescence phase. Virol J. 2020;17:125. PubMed https://doi.org/10.1186/s12985-020-01396-w

116. Lu S, Zhao Y, Yu W, Yang Y, Gao J, Wang J, et al. Comparison of nonhuman primates identified the suitable model for COVID-19. Signal Transduct Target Ther. 2020;5:157. PubMed https://doi.org/10.1038/s41392-020-00269-6

117. Xu L, Yu DD, Ma YH, Yao YL, Luo RH, Feng XL, et al. COVID-19-like symptoms observed in Chinese tree shrews infected with SARS-CoV-2. Zool Res. 2020;41:517–26. PubMed https://doi.org/10.24272/j.issn.2095-8137.2020.053

118. Shuai L, Zhong G, Yuan Q, Wen Z, Wang C, He X, et al. Replication, pathogenicity, and transmission of SARS-CoV-2 in minks. National Science Review. 2020;nwaa291. https://doi.org/10.1093/nsr/nwaa291

119. Mykytyn AZ, Lamers MM, Okba NM, Breugem TI, Schipper D, van den Doel PB, et al. Susceptibility of rabbits to SARS-CoV-2. Emerg Microbes Infect. 2021;10:1–7. PubMed https://doi.org/10.1080/22221751.2020.1868951

120. Bosco-Lauth AM, Hartwig AE, Porter SM, Gordy PW, Nehring M, Byas AD, et al. Experimental infection of domestic dogs and cats with SARS-CoV-2: pathogenesis, transmission, and response to reexposure in cats. Proc Natl Acad Sci U S A. 2020;117:26382–8. PubMed https://doi.org/10.1073/pnas.2013102117

121. Bertzbach LD, Vladimirova D, Dietert K, Abdelgawad A, Gruber AD, Osterrieder N, et al. SARS-CoV-2 infection of Chinese hamsters (*Cricetulus griseus*) reproduces COVID-19 pneumonia in a well-established small animal model. Transbound Emerg Dis. 2020;Sep 18:2020. PubMed https://doi.org/10.1111/tbed.13837

122. Chan JF-W, Zhang AJ, Yuan S, Poon VK-M, Chan CC-S, Lee AC-Y, et al. Simulation of the clinical and pathological manifestations of coronavirus disease 2019 (COVID-19) in golden Syrian hamster model: implications for disease pathogenesis and transmissibility. Clin Infect Dis. 2020;71:2428–46. PubMed https://doi.org/10.1093/cid/ciaa644

123. Sia SF, Yan L-M, Chin AW, Fung K, Choy K-T, Wong AY, et al. Pathogenesis and transmission of SARS-CoV-2 in golden hamsters. Nature. 2020;583:834–8. PubMed https://doi.org/10.1038/s41586-020-2342-5

124. Freuling CM, Breithaupt A, Müller T, Sehl J, Balkema-Buschmann A, Rissmann M, et al. Susceptibility of raccoon dogs for experimental SARS-CoV-2 infection. Emerg Infect Dis. 2020;26:2982–5. PubMed https://doi.org/10.3201/eid2612.203733

125. Richard M, Kok A, de Meulder D, Bestebroer TM, Lamers MM, Okba NM, et al. SARS-CoV-2 is transmitted via contact and via the air between ferrets. Nat Commun. 2020;11:3496. PubMed https://doi.org/10.1038/s41467-020-17367-2

126. Bao L, Deng W, Huang B, Gao H, Liu J, Ren L, et al. The pathogenicity of SARS-CoV-2 in hACE2 transgenic mice. Nature. 2020;583:830–3. PubMed https://doi.org/10.1038/s41586-020-2312-y

127. Hall JS, Knowles S, Nashold SW, Ip HS, Leon AE, Rocke T, et al. Experimental challenge of a North American bat species, big brown bat (*Eptesicus fuscus*), with SARS-CoV-2. Transbound Emerg Dis. 2020;Dec 9:2020. PubMed

128. Ulrich L, Wernike K, Hoffmann D, Mettenleiter TC, Beer M. Experimental infection of cattle with SARS-CoV-2. Emerg Infect Dis. 2020;26:2979–81. PubMed https://doi.org/10.3201/eid2612.203799

129. Suarez DL, Pantin-Jackwood MJ, Swayne DE, Lee SA, DeBlois SM, Spackman E. Lack of susceptibility to SARS-CoV-2 and MERS-CoV in poultry. Emerg Infect Dis. 2020;26:3074–6. PubMed https://doi.org/10.3201/eid2612.202989

130. Munster VJ, Feldmann F, Williamson BN, van Doremalen N, Pérez-Pérez L, Schulz J, et al. Respiratory disease in rhesus macaques inoculated with SARS-CoV-2. Nature. 2020;585:268–72. PubMed https://doi.org/10.1038/s41586-020-2324-7
